# Alternative RNA Conformations: Companion or Combatant

**DOI:** 10.3390/genes13111930

**Published:** 2022-10-23

**Authors:** Payal Gupta, Rushikesh M. Khadake, Shounok Panja, Krushna Shinde, Ambadas B. Rode

**Affiliations:** Regional Centre for Biotechnology, NCR Biotech Science Cluster, 3rd Milestone, Faridabad—Gurugram Expressway, Faridabad 121001, India

**Keywords:** RNA conformational ensemble, G-quadruplex, pseudoknot, riboswitch, gene regulation

## Abstract

RNA molecules, in one form or another, are involved in almost all aspects of cell physiology, as well as in disease development. The diversity of the functional roles of RNA comes from its intrinsic ability to adopt complex secondary and tertiary structures, rivaling the diversity of proteins. The RNA molecules form dynamic ensembles of many interconverting conformations at a timescale of seconds, which is a key for understanding how they execute their cellular functions. Given the crucial role of RNAs in various cellular processes, we need to understand the RNA molecules from a structural perspective. Central to this review are studies aimed at revealing the regulatory role of conformational equilibria in RNA in humans to understand genetic diseases such as cancer and neurodegenerative diseases, as well as in pathogens such as bacteria and viruses so as to understand the progression of infectious diseases. Furthermore, we also summarize the prior studies on the use of RNA structures as platforms for the rational design of small molecules for therapeutic applications.

## 1. Introduction

**Figure 1:**
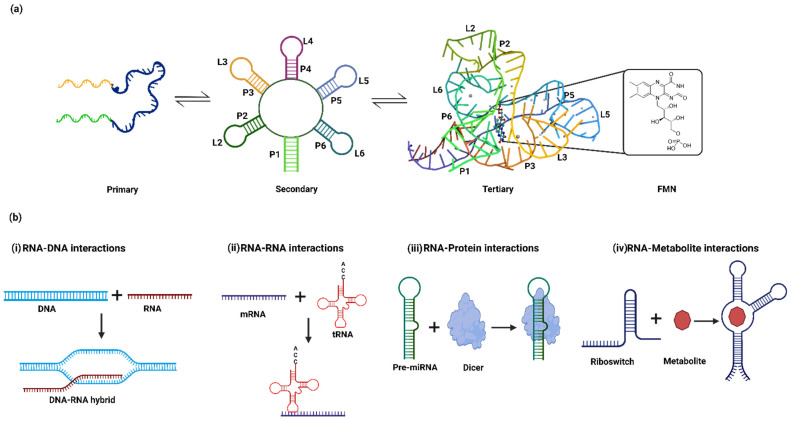
(**a**) The hierarchical folding of aptamers in a flavin mononucleotide (FMN) riboswitch. The primary structure of the FMN aptamers involves a single-stranded nucleotide sequence that sequentially folds into the secondary structure consisting of 6 stems (P1–P6) and 5 loops (L2–L6). The FMN binding pocket is formed in a tertiary structure via loop–loop (L2–L6 and L3–L5) and loop–helix (L6-P2 and L3-P5) interactions (PDB ID: 3F2Q). (**b**) Mode of action of regulatory RNA via interaction with (i) DNA (e.g., R-loop formation), (ii) other RNAs (tRNA riboswitch), (iii) proteins (e.g., dicer) and (iv) metabolites (e.g., FMN riboswitch).

Over the past decades, numerous studies on RNA have highlighted the regulatory roles of diverse RNA structures in different organisms. Besides serving as genetic material in viruses [1], it has several other regulatory roles in viruses [2], bacteria [3,4] and humans [5]. The regulatory role of RNA in cellular processes such as transcription, translation, splicing and nuclear export relies on its complex three-dimensional structures. RNA folds from a primary non-functional state to a tertiary functional state in a hierarchical manner (Figure 1a). During its folding, it can adopt alternate secondary and tertiary conformations in the biological systems due to sequence degeneracy [6]. The alternate secondary RNA conformations include helices, junctions, G-quadruplexes and hairpins formed via non-covalent interactions such as hydrogen bonding, Van der Waals forces and stacking interactions [7,8]. On the other hand, the tertiary structures are formed via interactions between the RNA secondary structures and diverse motifs such as loops, kissing-loops, base triples, pseudoknots, T-loop motifs, loop–helixes and A/G minor motifs [9,10].

RNA folding is dynamic in nature and has been very well studied using the free energy landscape model of the transactivation response (TAR) element of human immunodeficiency virus-1 (HIV-1) [11,12,13]. The dynamic ensemble of RNA occurs at the timescale of picoseconds to microseconds. The formation of alternate structures is governed by various cellular factors, which influence the conformational ensemble for biological functions. RNA conformers regulate biological functions via interactions with DNA [14,15], RNA [16,17], metal ions [18,19], metabolites [20] and proteins (Figure 1b) [21,22]. For example, MEG3 (chromatin-interacting long non-coding RNA) forms an RNA–DNA triplex structure that modulates the TGF β-pathway [15]. Other example involves RNA–RNA interactions, i.e., messenger RNA (mRNA) interacting with transfer RNA (tRNA), which are crucial for protein synthesis [23]. The interaction of Epstein–Barr virus nuclear antigen 1 (viral protein)-linking regions LR1 and LR2 with the RNA G-quadruplex regulates viral replication and episome maintenance is another interesting example [21]. Studies have shown that the RNA hairpin in the 3′ untranslated region (UTR) of Dengue viruses interacts with helicase DDX6 to mediate G1 phase arrest [22]. The precursor microRNA (Pre-miRNA) interacts with the Dicer protein to form mature miRNA [24,25]. The metalloregulatory riboswitches in bacteria recognize divalent metal ions such as cobalt (Co^2+^) or nickel (Ni^2+^) to regulate expression levels of metal transporters [18]. Winkler et al. reported that the flavin mononucleotide (FMN) binds to the conserved aptamer domain in the 5′ UTR of the mRNA-encoding genes responsible for riboflavin biosynthesis. The FMN riboswitch regulates the expression of metabolite biosynthetic genes in a metabolite-concentration-dependent manner [26].

**Figure 2:**
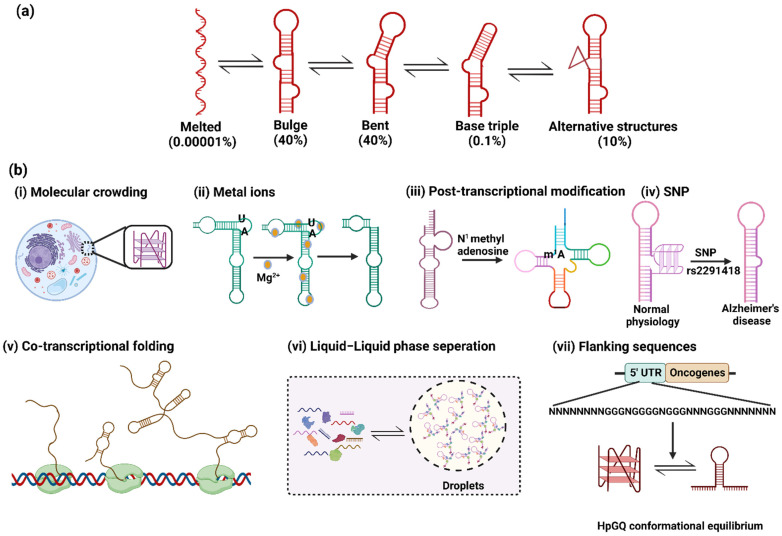
(**a**) HIV TAR RNA conformation ensemble consisting of diverse secondary structures with their respective populations. (**b**) Factors affecting the conformation ensemble: (i) molecular crowding in cells facilitates RNA folding in different conformations such as G-quadruplexes; (ii) metal ions like Mg^2+^ are required for ribozyme functioning to disrupt AU base pairing and the exposure of catalytic domains; (iii) post-transcriptional modifications such as N^1^ methyl adenosine regulate the proper folding of tRNA; (iv) the single-nucleotide polymorphism in miRNA 1229 leads to a conformational shift towards the hairpin structure, leading to Alzheimer’s disease; (v) co-transcriptional folding of RNA to form secondary structures such as hairpins; (vi) the liquid–liquid phase separation in RNA granules maintains the compartmentalization responsible for gene regulation; (vii) conformational equilibria between hairpin and G-quadruplex structures due to flanking sequences in cancer cells.

The competitive RNA conformers have individual functional roles, where one conformer leads to normal physiological function while the alternate conformer can lead to the disease condition. This could be better understood in the case of the intramolecular hairpin–G-quadruplex’s (HpGQ) conformational equilibria in proto-oncogenes, wherein the shift towards the G-quadruplex can lead to normal development, whereas the shift towards the hairpin can lead to the overexpression of proto-oncogenes [27], leading to cancer progression. This clearly suggests the need for a better understanding of the conformational equilibrium. The recent techniques and studies have shown that even the lowly populated structures of RNA from the conformational ensemble are equally important and play an important role in exerting the biological functions of RNA (Figure 2a) [28]. However, the majority of the studies have focused on the high-population-forming structures in the dynamic ensemble. Targeting the alternate RNA conformers using small molecules can serve as a potential therapeutic strategy; hence, detailed knowledge about diverse RNA conformers would help researchers in designing ligands that could target the specific RNA conformers. Herein, we review the studies that have focused on intramolecular RNA conformers, which play an important role in normal development and diseases. Our review particularly focuses on four competitive RNA conformations, which include hairpins, G-quadruplexes, pseudoknots and riboswitches. 

## 2. Dynamic Ensemble of RNA

RNA is a highly flexible and dynamic molecule in nature; it has the inherent ability to adopt inter-converting conformations (Figure 2a). This results in the collection of differently structured RNA populations (differing in folding states), referred to as ‘ensemble’. The dynamic ensembles of RNA conformations have been thoroughly studied and characterized using HIV TAR as a model system using techniques such as the fragment assembly of RNA with full-atom refinement–nuclear magnetic resonance (FARFAR-NMR) [11], NMR residual dipolar coupling (NMR-RDC) [12] and molecular dynamics simulations with NMR RDC (MD-NMR-RDC) [13]. The secondary stem–loop structure of TAR RNA is thermodynamically more stable than its tertiary structures. The tertiary structures have a relatively shorter life span–in the range of picoseconds to microseconds—than that of the secondary structure. The tertiary structures include bulge formations via the binding of the trans-activator of transcription (Tat) protein, causing coaxial stacking of the stems [11,29]. The partial stacking between two stems of bulge structures forms the bent structure [30]. In the later stage, the unstacking of three nucleotides of the bulge forms the base triple structure [12]. Other alternative secondary structures are also formed in the RNA ensemble with low abundance populations (Figure 2a) [28]. Therefore, it is becoming increasingly important to improve our understanding of the factors affecting these dynamic ensembles, which in turn regulate the different functions of RNA in cells.

### 2.1. Factors Affecting the Dynamic Ensembles of RNA

The conformation of the RNA can be affected by diverse cellular factors such as molecular crowding, metal ions, metabolites, proteins, chemical modifications, co-transcriptional folding, liquid–liquid phase separations and single-nucleotide polymorphisms (SNPs) (Figure 2b). We briefly discuss the above-mentioned factors here.

#### 2.1.1. Molecular Crowding 

The intra-cellular environment is always found to be crowded with a variety of biomolecules and salts up to concentrations of 300–400 mg/mL [31]. Molecular crowding affects not only the structures but also the functions of biomolecules, including RNA, via the excluded volume effect and by changing the hydration state. Thus, it is important to investigate the effects of crowding on RNA conformations to understand the ongoing RNA folding inside the cells [32]. 

To mimic the intracellular crowding conditions for in vitro studies, co-solutes such as polyethylene glycol (PEG), dextran and Ficoll have been widely used [32]. The high molecular weight crowding agent, e.g., PEG, has been found to stabilize the tertiary structure of RNA, i.e., the G-quadruplex, over secondary structure, i.e., the hairpin, through excluded volume effects [33,34]. Other than its role in modulating the structural conformations, crowding also affects other bio-molecular functions, e.g., it enhances the catalytic activity of ribozymes [35,36,37] and also increases the binding affinities of riboswitches with its respective metabolites [38]. 

#### 2.1.2. Metal Ions

Metal ions are important components of cellular constituents, which function as co-factors for multiple catalytic activities. These metal ions have a deep impact on RNA folding and interactions [39,40,41]. Divalent cations, mainly Mg^2+^, play an important role in the proper folding and functionality of RNAs [42]. The negatively charged backbone of RNA molecules severely affects the process of folding, and these electrostatic repulsions are neutralized by the counter-ions in the solution [43]. Both diffused and chelated ions have profound effects on the process of RNA folding into its tertiary structures, where diffused ions accumulate near the RNA due to electrostatic interactions, while chelated ions directly contact specific locations on the RNA [44]. The magnesium ions have an extended impact on the riboswitch functionality. A recent study showed that magnesium plays a key role in triplex formation during the pre-organization of the SAM-II riboswitch and in pseudoknot formation in the SAM-I riboswitch; this process of pre-organization allows the rapid detection of the ligand [45,46]. Magnesium also plays a key role in the catalytic function of ribozymes, where in absence of magnesium the ribozyme is found in its secondary structure, while upon the addition of magnesium, the A-U base pairing is destabilized to form a bend required to expose the catalytic domains [47]. 

In addition to divalent cations, monovalent cations, mainly Na^+^ and K^+^, facilitate functional RNA folding. The monovalent cations are well studied in the context of RNA G-quadruplexes; it is reported that the thermal stability of RNA G-quadruplexes is directly proportional to the salt concentration (Na^+^ and K^+^) [48].

#### 2.1.3. Co-Transcriptional Folding

The nascent RNA folding begins immediately after its synthesis by polymerase; this process of sequential folding during transcription is termed co-transcriptional folding. The co-transcriptional folding pathway is dependent on the rate of transcription and the population of transcription pausing sites [49,50]. More often the co-transcriptional folding enables the formation of metastable RNA structures such as hairpins, which are energetically more favorable than the complex ones such as G-quadruplexes and riboswitches. The impacts of the metastable RNA structural elements will vary depending on various kinetic factors such as the rates of transcription, the transition of the metastable structures and the translocation and degradation of the RNAs [51]. In kinetically regulated riboswitches, the ligand-binding rate is slower than the transcription rate; thus, a higher ligand concentration is required to reach equilibrium in vivo than for the ligand-binding affinity [26,52]. This change in activation threshold majorly depends upon co-transcriptional folding. The fluoride sensing aptamer of *Bacillus cereus* folds in identical structures, but the conformations from the dynamic ensemble differ in their stability. The ligand-free ensemble has a low abundance and a short life. This two-state ensemble shows the dependence of the riboswitch function on the transcription rate [14,53]. 

In addition to riboswitches, the co-transcriptional folding rates also influence the structural dynamics of RNA G-quadruplexes. The co-transcriptional folding rate of the RNA G-quadruplex is relatively slower than that of its competitive hairpin structures. Thus, the process of G-quadruplex formation is influenced by the kinetically favored metastable RNA structures. Although G-quadruplexes are thermodynamically stable, the G-quadruplex in the ORF of the *Escherichia coli* (*E. coli*) EutE gene did not show any change in the level of protein expression. The possible explanation for this is the rapid turnover of mRNAs in the organisms with relatively short lifecycles, as mRNAs get trapped in kinetically favored structures and are translated before the transition to the G-quadruplex [51]. 

#### 2.1.4. Post-Transcriptional Modifications

The cellular RNAs are post-transcriptionally modified to improve the stability of the different conformations and to facilitate the associated biological functions. The increase in stability of one conformer over the other by post-transcriptional modifications leads to the redistribution of RNA conformational ensembles [54]. The redistribution in the conformational ensemble can modulate RNA functions via different ribosomal binding proteins (RBPs) [55,56]. For example, N6 methyladenosine can destabilize the RNA helix interactions, thereby increasing the binding affinities of RBP to their target. On the other hand, the post-transcriptional modifications can also inhibit the RNA–protein interactions via alterations in the ensemble of non-canonical A-G mismatches ahead of the conformations required for their proper folding [57,58,59,60]. In contrast, N1 methyladenosine facilitates the proper folding of tRNA by destabilizing the base pairing in the native conformation [61]. The second major post-transcriptional RNA modification is uridine isomerization, which also has a stabilizing effect on the structure of the tRNA, and it has been suggested that the increase in stability is due to improved stacking or additional hydrogen bonding from pseudo-uridine [62,63,64]. 

#### 2.1.5. Liquid–Liquid Phase Separations

The liquid–liquid phase separation is an important phenomenon, whereby specific RNAs, proteins and other molecules are organized into separate granules [65,66]. These granules are responsible for regulating the gene expression by means of compartmentalization and by increasing the specific biomolecule concentration through altering the reaction specificities and kinetics [65,67,68]. RNA granules are phase-separated RNA molecules containing multivalent RNA–RNA and RNA–protein interactions [68,69]. In the structural context, it has been suggested that RNA molecules with distinguished functions but similar interacting partners forming different secondary structures are separated into distinct RNA granules [70], whereas those interacting non-specifically are excluded from the granules [71]. This phase separation indeed creates a microenvironment that affects the RNA ensemble due to the redistribution of folding energies. For example, the activity of the hammerhead ribozyme increases by 70-fold in phase-separated granules due to the stabilization of the folded RNA conformations [72]. It has been shown that in case of neurological diseases, the formation of RNA granules can be inhibited by means of RNA intercalating agents to stabilize or destabilize particular RNA secondary structures in a conformational ensemble [73]. However, there is a need for a deeper insight into the process of RNA granule formation to understand the RNA structure–function relationship.

#### 2.1.6. Single-Nucleotide Polymorphisms (SNPs) 

SNPs are the most common type of genetic variations; whereby single nucleotide changes occur at a specific location in the genome. Similar to other RNA regions, the 5′ and 3′ UTRs of mRNAs can adopt an ensemble of conformations [74,75,76]. The RNA nucleotide sequence plays an important role in defining the ensemble for a particular molecule. Thus, the single-nucleotide mutations leading to changes in the nucleotide sequence will affect the structural ensemble of the RNA by rearranging the base pair as well as other RNA interactions. The majority of single-nucleotide mutations in RNA have a local effect on the ensemble; however, a small subset of mutations imparts a profound and global effect [75]. A genome-wide association study was conducted to map mutations involved in the disease conditions. In this study, it was found that the human genome contains disease-associated SNPs that significantly alter the global conformation of UTRs to which they belong [75]. The miRNA maturation is highly sensitive to sequence variations that change the topology of the miRNA’s network of transient structures. The SNP in miRNA 125a interferes with the maturation of the miRNA by altering the RNA conformations [77].

#### 2.1.7. Competition between Alternative RNA Conformers

The majority of in vitro RNA structure–function studies are performed with respect to a stretch of a nucleotide sequence that is solely responsible for the formation of that structure. However, this is not true in cells where the upstream as well as downstream nucleotide regions play an important role in RNA folding. Thus, while studying the RNA conformational ensemble, it is important to include the adjacent nucleotide regions that are present in the natural context to understand the structure–function relationship in the cells. The effects of the sequence context on the RNA conformation have been investigated by studying the intramolecular conformation equilibria between G-quadruplex and hairpin structures. Bugaut et al. demonstrated that the adjacent sequence present in the context of the G tracts results in the formation of inter-converting HpGQ structures, which suggests that the intramolecular conformational equilibria between these structures strongly depends on the relative amounts of monovalent and divalent cations present in the solution [48,78]. The ATE 1 gene splicing was found to be regulated by the formation of multiple competing RNA structures [79]. In neurodegenerative diseases, the function of the polycomb repressive complex 2 (PRC2) depends on its recognition of the G-quadruplex structure. Thus, the presence of the competitive hairpin structure in equilibrium with the G-quadruplex affects the PRC2 function [80]. The conformational equilibrium shift between the hairpin–G-quadruplex structures in miRNA 1229 is responsible for the prognosis of Alzheimer’s disease [81]. Given the role of alternate RNA conformations in normal development and diseases, these structures are emerging as potential therapeutic targets [25,27,82,83].

## 3. RNA Alternate Conformers and Their Role in Diseases

In certain RNAs, non-canonical structures are present, which regulate cellular functions. Alterations in RNA conformers can contribute to normal development or disease conditions. Here, we mainly discuss four RNA conformers, i.e., HpGQ conformational equilibria from proto-oncogenes, G-quadruplex-protein interactions present in neurodegenerative diseases and pseudoknots and riboswitches from viruses and bacteria, respectively.

### 3.1. RNA Conformers in Cancer

**Figure 3:**
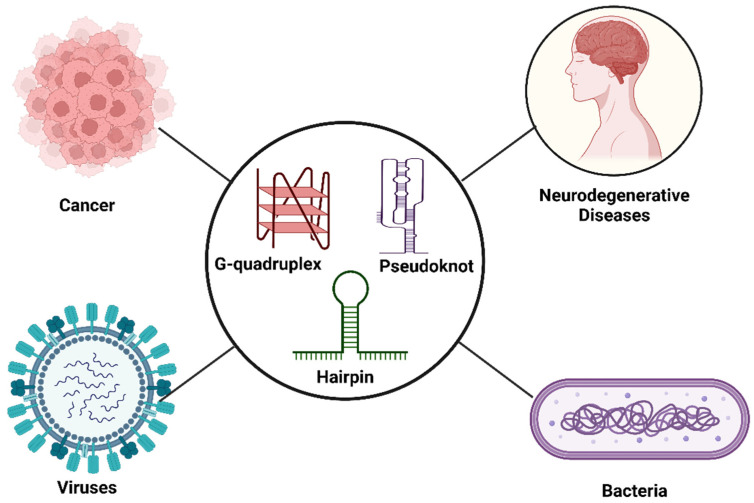
RNA conformers involved in cancer: neurodegenerative, viral and bacterial diseases.

Cancer, an uncontrolled growth of cells that invade the surrounding tissues and cause the tissue impairment, is one of the leading causes of death worldwide [84,85]. The RNA conformers regulate the biological functions, such as the gene expression, miRNA maturation, RNA interference and telomerase activity, suggesting their use as therapeutic targets (Figure 3). One of the RNA conformers, i.e., the G-quadruplex, has been suggested to regulate the cancer genes. Recently, it was shown that the G-quadruplex folding depends on the formation of a competitive hairpin structure, and the conformational equilibria between these two conformers plays an important role in cancer development [78]. Furthermore, the hairpin–G-quadruplex’s (HpGQ) conformational equilibria present in certain proto-oncogenes govern the gene expression [27,78,86]. The HpGQ conformational equilibria are also found in pre-miRNA, regulating the Dicer-mediated maturation of miRNA [25,83]. We have included unpublished data that include predicted putative HpGQ sequences in the 5′ UTR of human proto-oncogenes using the ENSEMBL genome browser [87] (Table 1). The HpGQ sequences are shortlisted on the basis of high G-score indicating stable G-quadruplex structures and high −Δ*G*° values suggesting the stability of the hairpin. To predict the G-score and −Δ*G*°, the QGRS mapper [88] and RNA folds [89] were employed, respectively.

In addition to the hairpin and G-quadruplex, other alternate conformers also regulate gene regulation in cancer-causing genes. For example, the human telomerase contains a pseudoknot structure that is essential for telomerase activity, which is upregulated in more than 90% of cancer cell lines [90]. The triplex structure formed due to the interaction of MIR100HG and p27 promotes cell proliferation in triple-negative breast cancer through RNA–DNA interactions [91]. 

### 3.2. RNA Conformers in Neurodegenerative Diseases

RNA-binding proteins related to the nervous system are widely studied, and any alteration in RNA conformers leads to normal brain development or neurological diseases [92] (Figure 3) such as frontotemporal dementia (FTD), amyotrophic lateral sclerosis (ALS), fragile X syndrome (FXS), Parkinson’s disease and Alzheimer’s disease. Conlon et al. predicted that the hnRNP H binding to the RNA G-quadruplex forming expanded *C9ORF72* repeats results in exon skipping and disruptive splicing, leading to the accumulation of hnRNP H/RNA G-quadruplex aggregates in the brain and causing ALS/FTD [93]. In another study, it was observed that a competition of formation between hairpin and G-quadruplex structures depends on the length and number of repeats. Therefore, in case of a reduced number of repeats, the G-quadruplex structure is formed, which binds with the polycomb-repressive complex 2 (PRC2) protein and sequester its functions, such as epigenetic regulation [80]. The RNA G-quadruplex in the 5′ UTR region of VPS35 and PRKN binds with guanine nucleotide binding protein like-1 (GNL-1), causing dysregulation of the genes associated with Parkinson’s disease [94]. The nucleolin protein recognize RNA G-quadruplex sequences with long loop lengths more efficiently as compared to short loop lengths [95], altering the biogenesis of the ribosomal RNA (rRNA) and causing ALS/FTD [96]. SNPs cause conformational shifts from G-quadruplex to hairpin structures in pre-miRNA 1229, thereby increasing the level of mature miRNA 1229 and subsequently the translation of the sortilin-related receptor 1 (SORL1) gene, causing Alzheimer’s diseases [81].

### 3.3. RNA Conformers in Viruses

Viruses replicate using the cellular machinery of the host organism. They can adopt various RNA conformations that regulate biological functions (Figure 3). Recently, the severe acute respiratory syndrome corona virus-2 (SARS-CoV-2) outbreak leads to the coronavirus disease, which has affected the lives of millions of individuals around the globe. Studies have suggested the presence of alternative conformers in the corona virus-2 genome that regulate the various functions of the coronaviruses. One of the most studied RNA conformers in COVID-19 is the pseudoknot formation. The frameshift element (FSE) forms an alternative pseudoknot structure in open reading frame (ORF) 1 that increases the frameshift rate to produce ORF1a and ORF1b from two overlapping frames [97,98]. The formation of the pseudoknot in the FSE also facilitates the viral replication [97,99,100]. The 3′ UTR region of coronaviruses contains stem–loop and pseudoknot structures, which regulate the RNA synthesis pathway [101]. The presence of these alternate pseudoknot conformers is not just limited to the coronaviruses, but there are several other virus families where pseudoknot structures are involved in the survival and virulence of the viruses. For example, outbreaks of the influenza A virus are due to SNPs causing virulence in the H5N1 strain due to a disruption of the equilibrium between hairpin and pseudoknot structures [102]. The Zika virus RNA contains multi-pseudoknot structures, making it resistant to cellular exonuclease in the host’s infected cells [103]. 

The other alternate RNA structure that plays functional roles in viruses is the G-quadruplex structure. The G-quadruplex structures are present in a wide variety of viruses, including HIV [104], Nipah virus [105] and SARS-CoV-2 [106]. The RNA G-quadruplex formed in the U3 region of HIV enables reverse transcriptase switching of the template, facilitating recombination [104]. The stabilization of G-quadruplex structures in the coding region of the SARS CoV-2 nucleocapsid phosphoprotein inhibits its translation [107]. The endonuclease activity at the replicative stage of the influenza A virus is mediated by the hairpin–loop present at the 3′ arm of the viral RNA promoter [108]. The pseudoknot at the 5′ terminal of the T4 bacteriophage gene 32 mRNA interacts with the gene 32 protein, which results in translation suppression [109]. Therefore, targeting RNA alternate conformers will help to cure viral diseases.

### 3.4. RNA Conformers in Bacteria

Bacterial mRNA contains *cis*-regulatory elements in the 5′ UTR that regulate gene expression and are known as riboswitches [110,111]. As the name suggests, there is switching of the conformation to regulate gene expression based on the metabolite binding (Figure 3). Riboswitches are composed of two domains: the aptamer domain and the expression platform. The binding of the ligand to the aptamer domain causes conformational changes in the expression platform controlling the expression of the downstream genes. Various ligands bind to riboswitches, including enzyme co-factors such as S-adenosyl methionine (SAM) [112], thiamine pyrophosphate (TPP) [113] and FMN [114]; nucleotide precursors such as guanine [115] and adenine [116]; amino acids such as lysine [117]; and metal ions such as magnesium [118], cobalt or nickel [18]. The SAM-1 riboswitch from the *Xanthomonas campestris* binds to SAM and performs dual functions, such as regulating methionine synthesis and binding to the uncharged Met tRNA releasing the Shine–Dalgarno sequence for translation initiation [119]. The binding of guanine, hypoxanthine or xanthine to the guanine riboswitch regulates the transcription of genes involved in purine metabolism and transport [115]. L-lysine binds to the lysine-responsive riboswitch to control gene expression [117] and for metabolic flux control [120]. The magnesium ion regulates the switching between the aptamer and expression platform in SAM-I riboswitches [121]. 

In addition to the riboswitches, there are several other non-canonical RNA structures that control the gene expression in bacteria. For example, the RNA G-quadruplex in *E. coli* and *Pseudomonas aeruginosa* (*P. aeruginosa*) regulates gene expression [122]. The stem–loop structure at the 5′ terminal of *E.* *coli* can stabilize the mRNA [123]. The Rho-independent transcriptional termination of bacteria is mediated by hairpin structures [124]. 

## 4. Targeting RNA Alternate Conformers Using Small Molecules for Therapeutics Applications

RNA has traditionally been thought to simply carry genetic information from the DNA to the protein. The advanced studies on RNA structures and functions have demonstrated that the RNA acts as a gene regulator. The RNA structure is a bridge from the primary nucleotide sequence to the biological function. RNA misfolding or aberrant RNA structures caused by mutations or abnormal interactions with other biomolecules can lead to diseases such as cancer, neurological disease, cardiovascular diseases, chronic obstructive pulmonary disease, liver disease and asthma. Thus, the small-molecule-mediated RNA structure interventions can serve as a promising strategy to develop a new class of drugs. In this section, we will summarize small molecule interventions for the treatment of cancer, neurodegenerative diseases and viral and bacterial infections. 

### 4.1. Cancer

Certain proto-oncogenes consist of G-quadruplexes that are in conformational equilibrium with hairpins. The conformational equilibria of the G-quadruplexes can be modulated using small molecules. For example, hTERT transcripts contain several tracks of G-rich sequences capable of forming G-quadruplexes. In one such effort, a synthetic ligand that was a member of triazine series was synthesized and tested for its ability to stabilize the G-quadruplexes. As a result, the binding of the ligand to the G-quadruplexes impaired the splicing machinery and the altered splicing pattern completely inactivated the telomerase activity [125]. 

**Figure 4:**
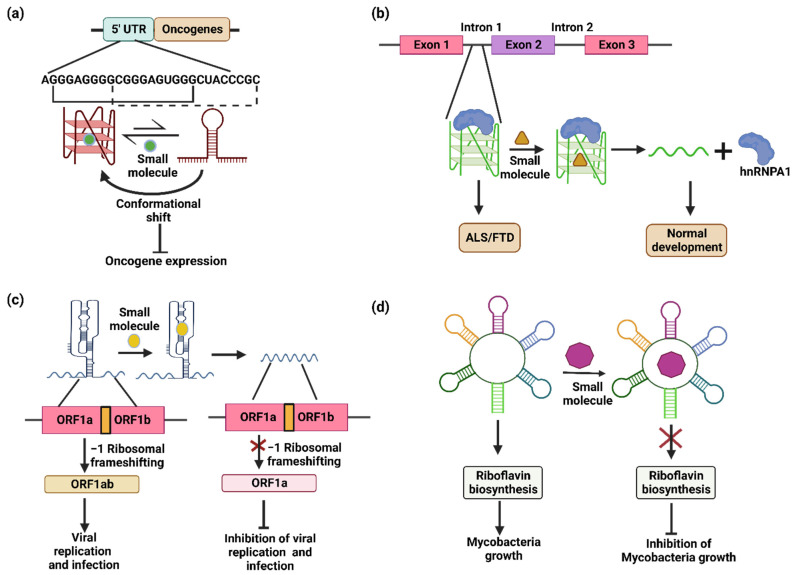
Targeting alternate RNA conformers using small molecules for therapeutics. (**a**) Small molecules shift the conformational equilibrium towards the G-quadruplex, causing suppression of the translation level in proto-oncogenes. (**b**) Intron 1 of the *C9ORF72* gene contains a G-quadruplex-forming sequence, sequestering the hnRNPA1 protein and resulting in ALS/FTD. Small molecules that destabilize the G-quadruplex release the hnRNPA1 protein in its free form leading for normal development. (**c**) The pseudoknot structure formed in the ORF1 leads to −1 ribosomal frameshifting and the formation of a complete ORF1ab product that causes viral replication and infection. The addition of small molecules disrupts the pseudoknot structure and prevents −1 ribosomal frameshifting, inhibiting viral replication and infection. (**d**) Small molecules targeting the FMN riboswitch inhibit the riboflavin biosynthesis pathway and subsequently inhibit the growth of mycobacteria.

Apart from telomerases, there are several other proto-oncogenes that possess putative G-quadruplex motifs. For example, RAS are proto-oncogenes encoding an intracellular signal transduction protein, functioning as molecular switches and regulating pathways responsible for proliferation and cell survival. The G-quadruplex motifs from the 5′ UTR of these RAS (NRAS and KRAS) mRNAs have been targeted by several ligands. For example, for the quinolinium derivatives RR82 and RR110 [126], the polyaromatic molecule RGB-1 [127] has been reported to suppress the expression of the luciferase and NRAS gene, respectively. The G-quadruplexes in KRAS are stabilized by alkyl derivatives of TMPyP4 [128] and anthrafurandiones [129]. The tetraphenylethene (TPE) derivatives have been shown to successfully modulate the conformational equilibria towards the G-quadruplex. In vitro translation assays demonstrated that the methyl derivative of tetra(4-pyridyl) tetraphenylethene (TPE−Py), i.e., TPE-MePy, suppressed the translation levels by 5.7-fold due to a shift in the conformational equilibria towards the G-quadruplex (Figure 4a) [27]. These results suggest that the small molecules affecting the RNA ensemble can be used as therapeutic drugs against diseases in which the RNA conformations are dysregulated.

### 4.2. Neurodegenerative Diseases

The RNA structures also play a key role in multiple degenerative diseases [130]. Frontotemporal dementia (FTD) and amyotrophic lateral sclerosis (ALS) are diseases caused due to repeat expansion in *C9ORF72*. The RNA from expanded repeats forms nuclear foci and undergoes repeat-associated non-ATG (RAN) translation. The expanded repeats adopt a hairpin structure in conformational equilibrium with G-quadruplexes. A study by Su et al. suggested that r(GGGGCC)exp-binding small molecules can be a potential therapeutic drug in cases of FTD/ALS. The r(GGGGCC)exp-binding small molecules can regulate the shift in conformational equilibria, resulting in a decrease in the RAN translation [131]. Another report by Simeone et al. observed similar results, whereby the frequency of the nuclear RNA foci and dipeptide repeat proteins was greatly reduced using three different chemical compounds with similar structural characters [132]. Zamiri et al. demonstrated that TmPyP4 can bind to r(GGGGCC)exp and distort the G-quadruplex, which ablates the interaction with hnRNPA1 and inhibits the translation of toxic dipeptides (Figure 4b) [133]. Other tri-nucleotide repeats of r(CGG) are found to play an important role in spinal muscular atrophy, where the expression of r(CGG) repeats generates pre-mRNA splicing errors in survival motor neuron 2 (SMN 2). A small molecule RG-7916 and its close analogs SMN-C2 and –C3 modulate the pre-mRNA splicing. The binding of the small molecules creates new functional binding sites that increase the binding of splicing modulators, highlighting the potential of small molecules to modulate pre-mRNA splicing [134]. The r(CGG)exp is also involved in fragile X-associated tremor/ataxia syndrome (FXTAS). The ligand 9-hydroxy-5, 11-dimethyl-2-(2-(piperidin-1-yl) ethyl)-6H-pyrido [22] carbazol-2-ium specifically binds and disrupts the lethal r(CGG)exp protein complex [135].

### 4.3. Viruses

Viruses are intracellular pathogens that thrive on host cells by hijacking the cellular machinery for their life cycle and sustainability. As RNA plays an important role in gene expression and virus replication, it can be used as a therapeutic target against viral diseases. SARS-CoV utilizes an essential ribosomal frameshifting signal pseudoknot (-1 RF) to synthesize key replication components (Figure 4c) [136]. The stability of the RNA structure is important for frameshifting and replication, which makes pseudoknot structures important therapeutic targets. Park et al. performed the computational screening of a library of compounds to target the RNA molecules. The hits were validated using in vitro and cell-based assays, and they reported a novel compound that decreases the efficiency of -1RF by 5-fold in HEK293 cells. The decrease in the conformational plasticity of the pseudoknot eventually hampers the function of RNA molecule [136]. A study on the HIV genome found an important dynamic ensemble of the HIV-1 TAR element RNA. This could be a prominent target for the inhibition of HIV replication [137]. Nentilimicin and 5-(N, N)-dimethylamiloride are the two compounds that bind the TAR with higher affinity, inhibit the binding of the natural partner Tat and impair the normal functions of the virus [138]. Hepatitis C virus (HCV), another RNA virus, uses the internal ribosomal entry site (IRES) to bypass the cap-dependent translation initiation [139]. The IRES sequences are prone to forming the stem–loop structures required for its function [140,141]. Benzimidazole-containing compounds were developed that possessed high binding affinities towards the IRES IIa and destabilized the stem–loop, which inhibited translation by inducing the conformational widening of the interhelical angle in the IRES [139]. In herpes simplex virus-1 (HSV-1), TMPyP4 was tested in infected cells, which stabilized the most abundant HSV-1 G-quadruplex present in the repeated regions of the viral genome. The compound neither influenced the HSV-1 entry nor the virus replication, but it induced the trapping of fully infectious HSV-1 virions in vesicles, independent of autophagy [142]. 

### 4.4. Bacteria

The emergence of multidrug resistance in bacteria is a real concern and a reason for researchers to seek new antibiotics. The different RNA conformations play an important role in the regulation of the metabolic pathways that are crucial for bacterial survival and virulence. The RNA conformations can be modulated by using small molecules to inhibit the bacterial growth. Thus, the RNA structures are promising targets for antibiotic development. The occurrence of the FMN riboswitch in a broad range of bacteria makes it an attractive target for antibacterial therapy. For example, the FMN riboswitch has been explored as an antibiotic target in Mycobacteria. The riboflavin biosynthesis is under the control of the FMN riboswitch in Mycobacteria and it lacks riboflavin transporters. Thus, the impairment of the riboswitch function using small molecules would stop the riboflavin synthesis in the mycobacteria, affecting its survival and growth. A library of riboflavin derivatives was synthesized and tested for anti-mycobacterial activity. The derivatives were found to significantly reduce the Mycobacterial growth (Figure 4d) [20]. Ribocil, a highly selective chemical modulator of the bacterial riboflavin riboswitch, acts as a structurally distinct mimic of the natural ligand FMN, represses riboswitch-mediated *ribB* gene expression and inhibits bacterial growth [143]. Another example is the TPP riboswitch, which regulates the expression of genes involved in the synthesis of TPP. Pyrithiamine is a structural analogue of TPP that is used to target the TPP riboswitch [144]. According to the literature, the riboswitches can be used as potential therapeutic targets [20,143,144]. There are multiple riboswitches present in human bacterial pathogens (Table 2) that can be targeted using synthetic derivatives of natural ligands. These bacterial pathogens were listed by the World Health Organization as Pathogens with an Immediate Requirement for Antibiotics [145] and by the Centers for Disease control and Prevention as Antibiotic-Resistant Threats [146] in 2017 and 2019, respectively. The Rfam database [147] was used to check the presence of various riboswitches in bacterial pathogens. 

## 5. Discussion

Although the focus of this review is on non-canonical structures and their regulatory role in human health, these structural conformations also play regulatory roles in animals and plants. Given the sequence similarities amongst the animals and humans, it is expected that the transcriptomes of animals could adapt non-canonical RNA conformations. Indeed, a bioinformatics study on genome sequences from animals such as chimpanzees, macaques, mice, rats and dogs suggested the presence of a conserved G-quadruplex in the 5′ UTR region of the NRAS, similar to that of humans [148], indicating its role in translation regulation. In addition, outbreaks of zoonotic pathogens cause fatal conditions in the animal population. Different RNA conformations in these pathogens can regulate their biological functions, meaning they can be used as drug targets. For example, the influenza A virus, which affects fowl animals, humans and other mammals, contains G-quadruplex-forming sequences in the regions encoding the polymerase complex protein, suggesting their possible role in viral replication [149]. The swine fever virus affecting the swine population contains an RNA pseudoknot structure upstream of the start codon that regulates the translation level by controlling the ribosome entry [150]. Apart from the animals, the presence of regulatory RNA conformers are also reported in plants, which regulate disease development and yield by controlling biological functions. For example, RNA G-quadruplexes in the transcriptome of Arabidopsis can control translation and modify plant growth [151]. The 3′ UTR of the thiamine biosynthetic gene (THIC) contains a TPP riboswitch in all plant species that controls splicing along with alternative 3′ end processing [152]. The pepino mosaic virus (PMV), which is threat to tomato production, contains pseudoknot structures in the 3′ UTR that overlap with the PolyA tail that is crucial for the replication of PMV RNA [153]. For more details, the roles of RNA conformers in plants are discussed in the articles by Liu et al. [154], Griffin et al. [155] and Yadav et al. [156].

The RNA conformers are located in the 3′ UTR [157,158,159], 5′UTR [160,161] and intronic regions [162,163] of RNA transcripts, by virtue of which they have profound effects on gene expression at the levels of transcription [164,165,166] and translation [164,167,168,169]. The specificity and spatiotemporal activity of non-canonical RNA structures make them important tools for controlling gene expression. RNA switches can control the level of protein expression in a concentration-dependent manner for specific ligand molecules [110]. A detailed understanding of the mechanisms utilized by various RNA conformers, such as G-quadruplexes and RNA pseudoknots, may lead to the development of novel genetic control elements. These genetic control elements can be implemented in the field of metabolic engineering to increase the production of metabolites [170,171] and pharmaceutical products [170], among others. The mRNA secondary structures were used by Pfleger et al. to improve the mevalonate titers in *E. coli*; the strain was further used to increase the production of an antimalarial drug artemisinin [170], while the synergetic effect of the lysine-ON and lysine-OFF riboswitches was used for improved lysine production in *Corynebacterium glutamicum* [172]. More details on the application of non-canonical RNA conformations for production improvements can be found in the comprehensive reviews by Zhang et al. [173], Schmidt et al. [174], Kang et al. [175] and Keasling et al. [176].

## 6. Conclusions and Future Perspective

The conformational RNA ensemble represents diverse RNA conformers with different biological functions. Since RNA conformations are indispensable for the regulation of biological functions, the disruption of RNA conformations can affect the normal development processes and can result in a disease condition, suggesting their importance as therapeutic targets. The use of small molecules has been a promising strategy; however, despite so much effort being put into targeting RNA conformers, the small of the molecules and their high specificity are still major limitations. The major challenges behind targeting RNA alternate conformers are as follows: (1) the dynamic conformational ensemble of the RNA; (2) the shorter life of RNAs in cells; (3) the non-specificity towards DNA and competitive RNA structures. Therefore, future studies on the development of ligands that can overcome the above-mentioned challenges will help researchers in the successful development of drugs for clinical usage. 

## Figures and Tables

**Table 1:** Putative HpGQ sequences in the 5′ UTR of human proto-oncogenes predicted using the ENSEMBL genome browser with the G-scores and stability values of the hairpin structure.

Gene	Sequence (5′–3′)	G-Score *	Hairpin Stability ** *−*Δ*G*° (Kcal/mol)
ABL2	CCACTCAGGGCCAGGGCCTGGGCTGGG	41	12.40
MN1	GGGCGGGGGGAGGGACCGCTC	41	8.80
RALB	GCCGCGGCTTGGGCAGGGTCAGGGCTGGG	41	9.90
MYBL1	CGGAGCCGGGCTGGGGCCAGGGCAGGG	41	9.20
SKI	CGGCGGCGGGGGCCGGGGGGGCCCGGG	40	11.90
HRAS	GGGTGGGGCCGGGCGGGGCCCGCG	42	14.40
ETS1	GGGAGCGGGCGAGGGCCGGGCAGGAGGAGC	41	7.30

ABL2: Abelson-related gene (tyrosine protein kinase); MN1: meningioma (transcription co-regulator); RALB: Ras-related protein B (Ras GTPase family); MYBL1: MYB proto-oncogene-like 1 (transcription factor); SKI: Sloan–Kettering Institute (transcription co-regulator); HRAS: Harvey rat sarcoma virus (Ras GTPase); ETS1: erythroblast transformation-specific factor 1 (transcription factor). * A higher G-score signifies a more stable G-quadruplex structure. ** A higher *−*Δ*G°* value signifies a more stable competitive hairpin structure.

**Table 2:** Riboswitches found in human bacterial pathogens.

Pathogenic Bacteria	Riboswitches								
	TPP	FMN	Cobalamin	Glycine	Lysine	Purine	Pre Q1 *	Mo Co-factor **	Mg ^2+^
*Mycobacterium tuberculosis*	+	+	+	+	−	−	−	−	−
*Acinetobacter baumannii*	+	+	+	+	−	−	−	−	−
*P. aeruginosa*	+	+	+	−	−	−	−	−	−
*Staphylococcus aureus*	+	+	−	+	+	+	−	−	−
*Neisseria gonorrhoeae*	+	−	−	+	−	−	+	−	−
*Salmonella*	+	+	+	+	+	−	−	+	+
*Enterococcus faecium*	+	+	−	−	+	+	+	−	−
*Streptococcus pneumoniae*	+	+	−	+	−	+	+	−	−
*Haemophilus influenzae*	+	−	−	+	+	−	+	+	−
*Shigella*	+	+	+	−	+	−	−	+	+

* Pre-queuosine. ** Molybdenum co-factor. + Presence of riboswitch. − Absence of riboswitch.

## Abbreviations

Common keywords used in the review with their definition.

Keyword	Definition
G-Quadruplex	G-quadruplex structures are formed by the guanine-rich regions via Hoogsteen base pairing.
Pseudoknot	A pseudoknot is an RNA structure that consists mostly of two helical segments joined by loops or single-stranded sections.
Riboswitch	Riboswitches are elements present in the 5′-untranslated region (UTR) of mRNAs that mediate cis-regulation over the transcript via specific binding with ligands.
RNA hairpin	A double-stranded RNA (dsRNA) stem and a terminal loop make up an RNA hairpin.
TAR RNA	TAR (transactivation response) RNA can be found in the 5′ end of all HIV-1 mRNAs that interact with cellular proteins to create ribonucleoprotein complexes.
Frontotemporal dementia	The term “frontotemporal dementia” (FTD) refers to a number of dementia types that involve the gradual degradation of the frontal and temporal lobes.
Amyotrophic lateral sclerosis	Upper and lower motor neurons degenerate in amyotrophic lateral sclerosis (ALS), which can cause muscle weakness and eventually paralysis.
Fragile X syndrome	A hereditary condition known as fragile X syndrome (FXS) is characterized by mild-to-moderate intellectual impairment.
hnRNP H	The heterogeneous nuclear ribonucleoprotein (hnRNP) H controls the maturation and post-transcriptional processing of pre-mRNA.
RAN translation	The translation of proteins that occurs without a start codon is known as repeat-associated non-AUG (RAN) translation.
